# Nutrition and health-seeking practices during pregnancy and lactation and potential strategies to increase micronutrient intakes among women in northern Lao PDR

**DOI:** 10.1017/jns.2022.94

**Published:** 2022-10-28

**Authors:** Taryn J. Smith, Dalaphone Sitthideth, Xiuping Tan, Charles D. Arnold, Sengchanh Kounnavong, Sonja Y. Hess

**Affiliations:** 1 Institute for Global Nutrition, University of California Davis, 3253 Meyer Hall, One Shields Avenue, Davis, CA 95616, USA; 2 Lao Tropical and Public Health Institute, Vientiane, Lao People's Democratic Republic

**Keywords:** Antenatal care, Breastfeeding, Food fortification, Iron–folic acid, Supplementation, Thiamine, Pregnancy, ANC, access to and utilisation of antenatal care, WHO, World Health Organization, IFA, iron–folic acid, Lao PDR, Lao People's Democratic Republic, TDD, thiamine deficiency disorders, SES, socioeconomic status, MUAC, maternal height, weight and left mid-upper arm circumference

## Abstract

Access to and utilisation of antenatal care (ANC) is important for optimising health and nutrition during pregnancy. This study aimed to assess adherence to and factors associated with ANC and antenatal supplement use among Laotian women, and consider culturally appropriate strategies to increase micronutrient intakes. Mother–child (aged 21 d to <18 months) dyads (*n* 699) enrolled in a hospital-based prospective cohort study with the community comparison group in Luang Prabang province were interviewed about their antenatal history, supplement use, household sociodemographic and dietary practices, including postpartum food avoidances. Ninety percent of women (mean age 24⋅7 ± 6⋅3 years) reported receiving ANC during their pregnancy, with the majority reporting four to seven contacts, while 84⋅6 and 17⋅3 % reported supplement use during pregnancy and lactation, respectively. Adequate ANC contacts (≥8) and supplement use was more likely among women with complete primary education and from higher socioeconomic status households, and less likely among women belonging to ethnic minority populations and those who delivered their child at home. All women continued to consume salt while adhering to postpartum food avoidances; however, 58⋅5 and 38⋅7 % of habitual consumers restricted fish and soy sauces, respectively. Eighty-six percent of women reported they would be willing to take supplements when adhering to postpartum dietary restrictions. Overall, women's reported ANC attendance and antenatal supplement use was suboptimal. Understanding predictors of and barriers to ANC and supplement use may help implement effective public health strategies to improve adherence. Alongside targeted supplementation, salt fortification with micronutrients may be a viable population-wide intervention that needs further evaluation.

## Introduction

The perinatal period presents a critical window of opportunity to reach pregnant and lactating women with several interventions that are essential to their own and their infant's health and well-being. Access to and utilisation of antenatal care (ANC) can improve women's experience of pregnancy and childbirth and prevent many pregnancy-related deaths and complications, which remain unacceptably high, especially in low-resource settings^(1)^. In 2016, the World Health Organization (WHO) updated recommendations on routine ANC for pregnant women^(2)^. These new guidelines recommend 8 ANC contacts, increased from 4 contacts, with the first occurring within the first 12 weeks of pregnancy and consists of 49 recommendations, including 14 nutritional interventions, encompassing counselling on a healthy diet and optimal nutrition, and preventive interventions such as micronutrient supplementation. Daily oral iron–folic acid (IFA) supplementation with 30–60 mg of elemental iron and 400 μg of folic acid is recommended for pregnant women to prevent maternal anaemia, low birth weight and preterm birth^(2)^. Although ANC coverage and utilisation has increased since the introduction of the WHO ANC model in 2002, globally, only 64 % of pregnant women received ≥4 ANC contacts throughout their pregnancy in 2007–2014^(2)^.

Lao People's Democratic Republic (Lao PDR), a predominantly rural lower-middle income country in Southeast Asia, has one of the highest maternal mortality rates in Southeast Asia at 185 per 100 000 live births^(3)^. The 2017 national survey reported that 62⋅2 % of pregnant women received ≥4 ANC contacts during their most recent pregnancy, while only 15⋅3 % received ≥8 ANC contacts and 17⋅9 % did not receive any ANC^(4)^. Additionally, the prevalence of anaemia among women aged 15–49 years was 39 %^(5)^, indicating a public health concern. In 2012, 52 % of women reported taking any iron supplements during their most recent pregnancy, and only one quarter took the recommended ninety doses or more^(6)^.

Thiamine deficiency disorders (TDDs), including the most severe form beriberi, presents a further public health concern in Lao PDR^(7–9)^ and other countries in South and Southeast Asia^(10,11)^ and is a contributor to infant mortality in the region^(9,12–14)^. Monotonous diets relying on thiamine-poor polished white rice, food insecurity, food preparation and cooking practices, and traditional highly restrictive postpartum diets place Laotian women at high risk of thiamine deficiency^(7)^. Thiamine deficiency has been observed in pregnant and lactating women who have increased demands for thiamine^(15,16)^ and could be an unrecognised complication of pregnancy and a preventable cause of maternal death^(17–19)^, with potential adverse effects on intrauterine growth and infant brain development^(20–23)^. Deficiency is rare in the neonatal period as thiamine levels are higher in newborns due to sequestration of thiamine *in utero*^(24,25)^. However, the risk is the highest in the first year of life, especially among predominantly breastfed infants of mothers who are thiamine deficient themselves, as thiamine content of breastmilk is related to maternal thiamine status^(26)^. It is, therefore, important to optimise maternal thiamine intakes and status during both pregnancy and lactation.

Recognising this risk, the Lao National Nutrition Strategy to 2025 and the Plan of Action 2016–2020^(27)^ and the Ministry of Health^(28)^ advises healthcare facilities to distribute thiamine supplements, in addition to IFA, to pregnant women in the second and third trimesters and for 3 months postpartum in high-risk areas. Furthermore, infants and children treated for beriberi or suspected TDDs in clinical settings are often provided with oral thiamine supplements following recovery and their mothers with 100 mg of oral thiamine twice daily for 30 d^(29)^. Currently, there are limited data on the coverage, adherence or impact of these supplementation programmes. Moreover, it is yet to be determined if supplements are culturally appropriate during the period of postpartum dietary restrictions. In addition to supplementation, micronutrient fortification of centrally processed food vehicles provides an opportunity for a large-scale, population-wide preventive intervention^(30)^. Given the high prevalence of postpartum food avoidances among Laotian women^(31,32)^, it is important to understand traditionally consumed and restricted foods and/or condiments when considering vehicles for food fortification.

The present study of mother–child dyads aimed to (1) assess the prevalence of ANC utilisation and women's report of supplement use during pregnancy and lactation and associated factors; (2) determine the reported consumption of thiamine supplements among infants and young children hospitalised with TDD-consistent symptoms and their mothers following discharge from the hospital and (3) explore if supplements and/or fortified condiments would be culturally appropriate strategies to improve micronutrient intakes among women adhering to culturally determined postpartum dietary restrictions.

## Methods

### Study design, setting and population

This is a secondary analysis of a hospital-based prospective cohort study with a community-based comparison group, implemented in Luang Prabang and surrounding provinces, northern Lao PDR, between June 2019 and January 2021 (ClinicalTrials.gov identifier NCT03626337). The primary objective of the study was to develop a case definition for thiamine-responsive disorders among infants and young children, for which the study protocol has previously been described in detail^(33)^. Briefly, infants and young children aged 21 d to <18 months with symptoms suggestive of TDDs and seeking care at the Lao Friends Hospital for Children in Luang Prabang were eligible for participation (Supplementary Table S1). A community cohort of infants and young children frequency matched by age, sex and village of residence were enrolled to serve as a comparison group. The frequency matching of the community cohort aimed to achieve a comparison group that had a similar distribution on the indicated variables as the hospital cohort to minimise confounding biases. To ensure a well-balanced match, characteristics of hospitalised children were summarised, and community children were enrolled on a weekly basis to avoid seasonal discordance. Prior to visiting the health district, the health centre of the selected village was contacted to assist with identifying a child of the right target age (within a 2-week period) and living in the selected village or, if not available, a neighbouring village in the catchment area of the health centre. Health centre staff or village heads established contact with the child's parents/caregivers and invited them for a study appointment at the health centre. All primary female caregivers of hospitalised and community children were invited for participation, providing that informed consent was obtained.

### Data collection

During the hospital stay and the study appointment at the health centre for the community cohort, women completed a broad range of questionnaires. Women were interviewed about their child's birth history, including the number of ANC visits during the pregnancy of the study child (verified with the antenatal book), place of delivery (at home, a health centre or a hospital) and maternal supplement use during pregnancy and lactation. Healthcare facilities, including provincial and district hospitals and health centres in Luang Prabang and surrounding provinces, were contacted to determine supplements that were distributed to pregnant women during ANC contacts, the dose and if they had recently changed the supplements distributed during ANC. Similarly, information was collected on supplements that were distributed during lactation, the dose and the duration (in months) that postnatal supplements were provided. In some instances, women did not know what supplements they took during pregnancy and/or lactation but knew the health facility that distributed the supplements to her. In these cases, the supplement type was assigned based on the information provided by the health facility.

As culturally determined restrictive postpartum diets are common in the study population, a comprehensive questionnaire was developed to interview women about their diets during pregnancy and postpartum, as previously described in detail^(32)^. This included the determination of what foods and condiments (salt, fish sauce and soy sauce) were consumed or restricted in weekly (for the first 4 weeks) and monthly intervals postpartum. Women were also asked if they would be willing to take supplements during this time if provided to them by health centres. Infant and young child-feeding practices were assessed based on caregiver recall using structured survey questions of breastfeeding practices and, if applicable, consumption of foods^(34)^ in the previous 24 h for community children or day before going to hospital for hospitalised children; in case, breastfeeding or dietary intakes were affected by illness or hospitalisation. Self-reported indicators of socioeconomic status (SES) included education and occupation of the mother and household head, household size and composition, housing characteristics, access to utilities and household ownership of assets and land. These proxy indicators were used to estimate the household SES index using principal component analyses^(35)^. Food security was assessed using the Household Food Insecurity Access Scale^(36)^. Social desirability bias evaluated the accuracy of self-reported measures using five brief questions as previously proposed^(37,38)^. A social desirability score was created by adding up the number of socially desirable responses (0–2 = low score; 3 = medium score; 4 = high score; 5 = very high score)^(38)^.

Maternal height, weight and left mid-upper arm circumference (MUAC), child recumbent length and weight^(39)^, and maternal and child complete blood count using venous blood^(33)^ were assessed following standard protocols as previously described.

A follow-up phone interview was conducted with caregivers of hospitalised children 4 weeks after discharge from the hospital. Caregivers were asked if they had received thiamine supplements at discharge from the hospital for the child and the mother herself, if they had taken the supplements/given the supplements to the child, how often and, if not, the reason for not taking the supplements.

All data were entered electronically into Samsung tablets (Samsung Galaxy Tab. 3V, Seoul, South Korea) with the use of SurveyCTO (Dobility, Cambridge, MA, USA).

### Ethics

This study was conducted according to the guidelines laid down in the Declaration of Helsinki, and all procedures involving human participants were approved by the National Ethics Committee for Health Research, Ministry of Health, Lao PDR (Ref. 2018.115.Vie) and the Institutional Review Board of the University of California, Davis, USA (Ref. 1329444-3). Written or finger-printed informed consent was obtained from at least one primary caregiver for the child's participation in the study, and from the women for their own participation, after a detailed explanation of the study in a language appropriate to the family.

### Statistical analysis

A detailed statistical analysis plan was developed and published prior to analysis^(40)^. Baseline characteristics, prevalence of ANC utilisation and supplement use during pregnancy and breastfeeding, and reported thiamine supplement use after hospital discharge by children and their mothers are presented as mean ± standard deviation for continuous variables and frequencies (percentages) for categorical variables. Independent *t*-tests for continuous data and Pearson's chi-squared test for categorical data were used to compare differences between groups (hospital *v*. community cohorts; maternal ethnic groups).

Bivariate and multivariable logistic regression analyses were conducted among biological mothers to assess associations of ANC utilisation and supplement use with maternal (age, ethnic group, education, gravidity and delivery location) and household socioeconomic characteristics (household SES index and food insecurity access scale) while controlling for social desirability bias and whether women were interviewed at the hospital or in the community. First, potential factors associated with ANC and supplement use were evaluated using minimally adjusted bivariate logistic regression models. All predictors associated with the outcome at a level of *P-*value < 0⋅1 were then included in the multivariable models. Multicollinearity was assessed using the variance inflation factors. Results are presented as odds ratio (OR) and 95 % confidence interval (CI). All tests were two-sided at a 5 % level of significance.

Condiment consumption and supplement use while adhering to postpartum food restrictions were also summarised as frequency and percentage. The frequency of condiment consumption was categorised as 7 days a week, 5–6 days a week, 3–4 days a week, 1–2 days a week or never. Women who consumed salt, fish sauce or soy sauce for ≥3–4 days a week in general were considered habitual consumers. Dichotomous variables were defined for each condiment whether or not women had consumed the condiment at any time during the period of postpartum dietary restrictions. Statistical analyses were performed using Stata, version 14.2 (StataCorp, College Station, TX, USA).

## Results

### Participants’ characteristics

**Fig. 1.**
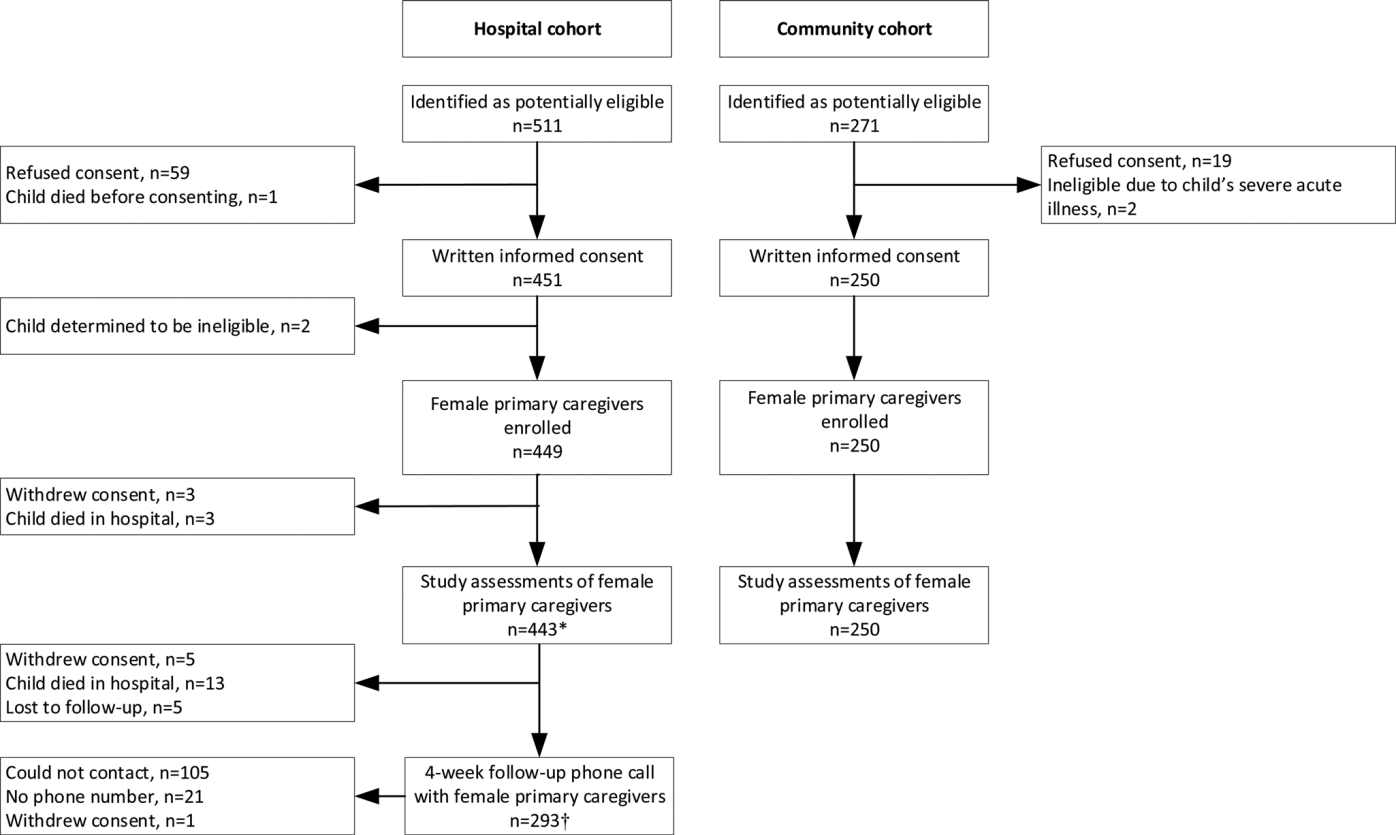
Flowchart of female caregivers’ eligibility, enrolment and data collection in the hospital and community cohorts. *Sample size for different assessments may vary. ^†^*n* 6 children died at home after discharge from the hospital; therefore, phone interview was not completed.

A total of 782 female caregivers and their children were identified as potentially eligible for participation in the study, of which 699 were enrolled (Fig. 1), the majority of which were biological mothers (98⋅7 %; Table 1). Among the hospital cohort, 293 caregivers were contacted for a follow-up phone call 4 weeks after the child's discharge from the hospital.

**Table 1.** Characteristics of women and children in the hospital and community cohorts^a^

Characteristics	All (*n* 699)	Hospital (*n* 449)	Community (*n* 250)	*P-*value
	*n* (%) or mean ± sd			
Women				
Age (years)	24⋅8 ± 6⋅5	24⋅8 ± 6⋅7	24⋅8 ± 6⋅2	0⋅99
Primary female caregiver				0⋅39
Biological mother	690 (98⋅7)	441 (98⋅2)	249 (99⋅6)	
Adoptive mother	4 (0⋅6)	3 (0⋅7)	1 (0⋅4)	
Grandmother	4 (0⋅6)	4 (0⋅9)	0 (0⋅0)	
Aunt	1 (0⋅1)	1 (0⋅2)	0 (0⋅0)	
Province of residence				0⋅23
Luang Prabang	638 (91⋅3)	411 (91⋅5)	227 (90⋅8)	
Oudomxay	39 (5⋅6)	21 (4⋅7)	18 (7⋅2)	
Xayaboury	13 (1⋅9)	8 (1⋅8)	5 (2⋅0)	
Other	9 (1⋅2)	9 (2⋅0)	0 (0⋅0)	
Ethnic group				<0⋅001
Lao	104 (15⋅1)	49 (11⋅1)	55 (22⋅0)	
Khmu	228 (33⋅0)	107 (24⋅3)	121 (48⋅4)	
Hmong	334 (48⋅3)	274 (62⋅1)	60 (24⋅0)	
Other	25 (3⋅6)	11 (2⋅5)	14 (5⋅6)	
Education				<0⋅001
No formal education	135 (19⋅6)	105 (23⋅9)	30 (12⋅0)	
Some/completed primary	215 (31⋅2)	128 (29⋅1)	87 (34⋅8)	
Some completed secondary	295 (42⋅8)	187 (42⋅5)	108 (43⋅2)	
College/university	45 (6⋅5)	20 (4⋅5)	25 (10⋅0)	
Occupation				<0⋅001
Does not work	9 (1⋅3)	4 (0⋅9)	5 (2⋅0)	
Housewife	142 (20⋅6)	75 (17⋅0)	67 (26⋅8)	
Farmer	439 (63⋅6)	315 (71⋅6)	124 (459⋅6)	
Unskilled labourer	4 (0⋅6)	3 (0⋅7)	1 (0⋅4)	
Skilled worker	96 (13⋅9)	43 (9⋅8)	53 (21⋅2)	
Gravidity^b^	2⋅6 ± 1⋅8	2⋅7 ± 2⋅0	2⋅3 ± 1⋅5	0⋅005
ANC contacts				<0⋅001
0–3	222 (33⋅0)	174 (40⋅9)	48 (19⋅3)	
4–7	284 (42⋅2)	171 (40⋅2)	113 (45⋅6)	
≥8	167 (24⋅8)	80 (18⋅8)	87 (35⋅1)	
Location of delivery of infant				<0⋅001
Home	177 (25⋅7)	133 (30⋅3)	44 (17⋅6)	
Health centre	284 (41⋅2)	161 (36⋅7)	123 (49⋅2)	
Hospital	195 (28⋅3)	131 (29⋅8)	64 (25⋅6)	
Other	33 (4⋅8)	14 (3⋅2)	19 (7⋅6)	
Household SES index^c^	0⋅0 ± 1⋅0	−0⋅1 ± 1⋅0	0⋅2 ± 1⋅0	<0⋅001
Food insecurity category^d^				<0⋅001
Moderate to severe	234 (33⋅9)	175 (39⋅8)	59 (23⋅6)	
Mild to none	456 (66⋅1)	265 (60⋅2)	191 (76⋅4)	
Social desirability score^e^				<0⋅001
Low	229 (33⋅1)	173 (39⋅2)	56 (22⋅4)	
Medium	304 (44⋅0)	153 (34⋅7)	151 (60⋅4)	
High	138 (20⋅0)	100 (22⋅7)	38 (15⋅2)	
Very high	20 (2⋅9)	15 (3⋅4)	5 (2⋅0)	
BMI (kg/m^2)^				0⋅39
<18⋅5	57 (8⋅5)	38 (8⋅9)	19 (7⋅6)	
≥18⋅5–24⋅9	523 (77⋅6)	334 (78⋅6)	189 (75⋅9)	
≥25⋅0–29⋅9	87 (12⋅9)	48 (11⋅3)	39 (15⋅7)	
≥30⋅0	7 (1⋅0)	5 (1⋅2)	2 (0⋅8)	
Height <150 cm^f^	363 (53⋅9)	254 (59⋅8)	109 (43⋅8)	<0⋅001
MUAC <23⋅5 cm^g^	286 (42⋅2)	190 (44⋅3)	96 (38⋅6)	0⋅15
Children				
Age (months)	4⋅3 ± 3⋅4	4⋅3 ± 3⋅4	4⋅5 ± 3⋅2	0⋅51
Male	417 (59⋅7)	273 (60⋅8)	144 (57⋅6)	0⋅41
Breastfeeding status^h^				<0⋅001
Exclusive breastfeeding	427 (61⋅2)	278 (62⋅1)	149 (59⋅6)	
Predominant breastfeeding	45 (6⋅4)	37 (8⋅3)	8 (3⋅2)	
Mixed milk feeding	44 (6⋅3)	33 (7⋅4)	11 (4⋅4)	
Continued breastfeeding	141 (20⋅2)	66 (14⋅7)	75 (30⋅0)	
No longer breastfeeding	41 (5⋅9)	34 (7⋅6)	7 (2⋅8)	
Stunted^i^	173 (26⋅0)	136 (32⋅7)	37 (14⋅8)	<0⋅001
Wasted^i^	52 (7⋅8)	47 (11⋅4)	5 (2⋅0)	<0⋅001
Underweight^i^	141 (21⋅2)	119 (28⋅6)	22 (8⋅8)	<0⋅001

ANC, antenatal care; BMI, body mass index; MUAC, mid-upper arm circumference; SES, socioeconomic status.

a Sample size for different assessments may vary; ANC contacts during pregnancy with the study child and gravidity are for biological mothers; anthropometry and haemoglobin were only assessed for biological and adoptive mothers.

b *n* 687; *n* 437 hospital; *n* 250 community.

c SES index derived from principal component analysis using self-reported measures of housing characteristics, household access to utilities and household ownership of assets and land^(35)^; *n* 687; *n* 439 hospital; *n* 248 community.

d Food insecurity assessed using the Household Food Insecurity Access Scale^(36)^.

e A social desirability score was created by adding up the number of socially desirable responses: 0–2 = low score; 3 = medium score; 4 = high score; 5 = very high score^(38)^.

f *n* 674; *n* 425 hospital; *n* 249 community.

g *n* 678; *n* 429 hospital; *n* 249 community

h Predominant breastfeeding defined as breastfeeding with certain liquids (water, water-based drinks, fruit juice); mixed milk feeding defined as breastfeeding with infant formula and/or animal milk^(34)^.

i *n* 666; *n* 416 hospital; *n* 250 community.

Characteristics of all female caregivers and their children are shown in Table 1. Specifically for the biological mothers (*n* 690), mean age was 24⋅7 ± 6⋅3 years and gravidity was 2⋅6 ± 1⋅8. For indicators of malnutrition among biological mothers, 8⋅5 % were underweight (9⋅0 and 7⋅7 % in the hospital and community cohorts, respectively; *P* = 0⋅55) and 42⋅4 % had MUAC <23⋅5 cm (44⋅6 and 38⋅7 % in the hospital and community cohorts, respectively; *P* = 0⋅14). Almost half of the enrolled women belonged to the Hmong ethnic group (48⋅3 %), followed by Khmu (33⋅0 %) and Lao (15⋅1 %) ethnic groups; however, this differed between the hospital and community cohorts (Table 1). Among all female caregivers, education, gravidity, household SES index and food insecurity and indicators of malnutrition (height <150 cm and MUAC <23⋅5 cm) significantly differed by the ethnic group (Supplementary Table S2). The mean social desirability score was 2⋅7 ± 1⋅1, with most women having low to medium scores. In the hospital cohort, more children were stunted, wasted and underweight compared to the community cohort (all *P* < 0⋅001).

### ANC utilisation and supplement use during pregnancy and lactation

Ninety percent of women received ANC during their pregnancy with the study child, with the majority reporting receiving 4–7 ANC contacts, and 69⋅5 % gave birth to the child at a healthcare facility (Table 1). However, this varied by the ethnic group with 51⋅5 % of women belonging to the Hmong ethnic group, reporting that they received 0–3 ANC contacts, while 46⋅4 % of Khmu women reported 4–7 ANC contacts and 47⋅1 % of Lao women reported ≥8 ANC contacts (Supplementary Table S2). Over 90 % of women belonging to the Lao ethnic group delivered their child at a healthcare facility, compared to 69⋅6 % of Khmu women and 61⋅2 % of Hmong women (Supplementary Table S2).

Ninety-seven healthcare facilities across six provinces in northern Lao PDR were interviewed about supplements distributed to pregnant and lactating women (Supplementary Table S3). The majority of healthcare facilities in Luang Prabang province reported distributing IFA and thiamine during both pregnancy (87⋅5 %) and lactation for 3 months postpartum (85⋅0 %). However, thiamine supplement distribution differed by province, with 29⋅4 and 41⋅2 % of healthcare facilities interviewed outside of Luang Prabang providing thiamine supplements to pregnant and lactating women, respectively, compared to 90⋅0 and 86⋅3 %, respectively, in Luang Prabang province.

Among women who reported supplement use, 166 (28⋅8 %) and 25 (21⋅2 %) women did not know what supplements they took during pregnancy and lactation, respectively, and supplement types were assigned based on the health facility supplementation information. Of the 681 biological mothers, 576 (84⋅6 %) women reported taking supplements during their pregnancy; most common supplement types were iron alone (41⋅7 %) and IFA (39⋅9 %) (Table 2). Despite almost 80 % of the health facilities interviewed reporting the distribution of thiamine supplements to pregnant women, only 36⋅6 % of women reported taking thiamine supplements during their pregnancy. Of women who reported taking supplements during pregnancy, the majority reported taking the supplements daily and for the duration of their pregnancy (Table 2). During lactation however, only 118 (17⋅3 %) women reported taking supplements; 10⋅1 % iron alone, 9⋅4 % thiamine and 5⋅6 % IFA (Table 2). Supplement use during pregnancy (73⋅9 %) and lactation (12⋅3 %) was lowest among women belonging to the Hmong ethnic group (Supplementary Table S2).

**Table 2.** Reported supplement use during pregnancy and lactation among biological mothers (*n* 681)

	Iron–folic acid	Iron alone	Folic acid alone	Thiamine
	*n* (%)			
Pregnancy^a^				
Reported supplement use during pregnancy	272 (39⋅9)	284 (41⋅7)	26 (3⋅8)	249 (36⋅6)
Duration of supplement use				
Whole of pregnancy	152 (55⋅9)	205 (72⋅2)	18 (69⋅2)	158 (63⋅5)
4–6 months	67 (24⋅6)	44 (15⋅5)	4 (15⋅4)	50 (20⋅1)
≤3 months	48 (17⋅6)	34 (12⋅0)	4 (15⋅4)	39 (15⋅7)
Unknown	0 (0)	1 (0⋅4)	0 (0)	2 (0⋅8)
Reported supplement consumption				
Daily	237 (87⋅1)	263 (92⋅6)	23 (88⋅5)	234 (94⋅0)
5–6 times a week	19 (7⋅0)	6 (2⋅1)	1 (3⋅8)	8 (3⋅2)
3–4 times a week	5 (1⋅8)	5 (1⋅8)	0 (0)	2 (0⋅8)
1–2 times a week	10 (3⋅7)	5 (1⋅8)	2 (7⋅7)	4 (1⋅6)
Less than once a week	0 (0)	4 (1⋅4)	0 (0)	0 (0)
Unknown	1 (0⋅4)	1 (0⋅4)	0 (0)	1 (0⋅4)
Lactation^b^				
Reported supplement use during lactation	38 (5⋅6)	69 (10⋅1)	0 (0)	64 (9⋅4)
Reported supplement consumption				
Daily	36 (94⋅7)	68 (98⋅6)	0 (0)	63 (98⋅4)
5–6 times a week	1 (2⋅6)	1 (1⋅4)	0 (0)	1 (1⋅6)
3–4 times a week	0 (0)	0 (0)	0 (0)	0 (0)
1–2 times a week	0 (0)	0 (0)	0 (0)	0 (0)
Less than once a week	1 (2⋅6)	0 (0)	0 (0)	0 (0)
Unknown	0 (0)	0 (0)	0 (0)	0 (0)

a During pregnancy, 65 (9⋅5 %) women reported that they did not know what supplements they took.

^b^During lactation, 8 (1⋅2 %) women reported that they did not know what supplements they took.

Associations between reported ANC contacts and maternal and household characteristics are shown in Table 3. In multivariable analyses, women with the complete primary education were 1⋅9 times more likely to report 4–7 ANC contacts (OR 1⋅89, 95 % CI 1⋅16, 3⋅06; *P* < 0⋅01) and 2⋅9 times more likely to report ≥8 ANC contacts (OR 2⋅88, 95 % CI 1⋅46, 5⋅67; *P* < 0⋅01) compared to women with incomplete primary education. Similarly, women who lived in households with higher SES were 1⋅8 times more likely to report ≥8 ANC contacts (OR 1⋅76, 95 % CI 1⋅21, 2⋅56; *P* < 0⋅01). Women were less likely to report ≥8 ANC contacts if they belonged to the Hmong ethnic group compared to Lao (OR 0⋅13, 95 % CI 0⋅05, 0⋅33; *P* < 0⋅001) and delivered their child at home compared to a hospital (OR 0⋅10, 95 % CI 0⋅04, 0⋅25; *P* < 0⋅001). Women with greater gravidity and those with greater household food insecurity were less likely to report ≥8 ANC contacts in the bivariate model, but this did not remain significant in the multivariable model.

**Table 3.** Factors associated with reported antenatal care contacts during pregnancy among biological mothers (*n* 681) in bivariate and multivariable models

Variables	Bivariate model				Multivariable model^a^			
	4–7 ANC contacts^b^		≥8 ANC contacts^b^		4–7 ANC contacts^b^		≥8 ANC contacts^b^	
	OR	95 % CI	OR	95 % CI	OR	95 % CI	OR	95 % CI
Maternal age	0⋅98	0⋅95, 1⋅01	1⋅00	0⋅96, 1⋅03				
Maternal ethnic group								
Lao	Ref.		Ref.		Ref.		Ref.	
Khmu	0⋅38	0⋅16, 0⋅87*	0⋅26	0⋅11, 0⋅60**	0⋅68	0⋅26, 1⋅73	1⋅01	0⋅37, 2⋅73
Hmong	0⋅14	0⋅06, 0⋅31***	0⋅04	0⋅02, 0⋅10***	0⋅26	0⋅11, 0⋅65**	0⋅13	0⋅05, 0⋅33***
Maternal education								
Incomplete primary	Ref.		Ref.		Ref.		Ref.	
Complete primary	2⋅88	1⋅98, 4⋅20***	6⋅58	3⋅92, 11⋅0***	1⋅89	1⋅16, 3⋅06**	2⋅88	1⋅46, 5⋅67**
Gravidity	0⋅79	0⋅71, 0⋅88***	0⋅70	0⋅60, 0⋅80***	0⋅92	0⋅81, 1⋅04	0⋅95	0⋅79, 1⋅13
Location of delivery								
Hospital	Ref.		Ref.		Ref.		Ref.	
Health centre	0⋅76	0⋅47, 1⋅25	0⋅56	0⋅33, 0⋅96*	0⋅59	0⋅31, 1⋅11	0⋅50	0⋅24, 1⋅03
Home	0⋅20	0⋅12, 0⋅33***	0⋅05	0⋅03, 0⋅12***	0⋅23	0⋅12, 0⋅45***	0⋅10	0⋅04, 0⋅25***
Other	0⋅89	0⋅31, 2⋅53	0⋅88	0⋅30, 2⋅64	0⋅92	0⋅28, 3⋅00	1⋅25	0⋅33, 4⋅71
Household SES index	1⋅72	1⋅39, 2⋅11***	2⋅73	2⋅15, 3⋅48***	1⋅19	0⋅87, 1⋅62	1⋅76	1⋅21, 2⋅56**
HFIAS	0⋅97	0⋅93, 1⋅00	0⋅90	0⋅85, 0⋅95***	1⋅02	0⋅97, 1⋅07	0⋅98	0⋅92, 1⋅05

ANC, antenatal care; CI, confidence interval; HFIAS, household food insecurity access scale; OR, odds ratio; SES, socioeconomic status.

a Variables with *P* < 0⋅1 at bivariate level were included in the multivariable logistic regression model.

b Compared to 0–3 ANC contacts during the pregncancy with the study child (reference).

* *P* < 0⋅05; **P < 0⋅01; ***P < 0⋅001.

Of the women who reported ≥8 and 4–7 ANC contacts, 99⋅4 and 95⋅8 % reported taking supplements during pregnancy, respectively, compared to 59⋅0 % who reported 0–3 ANC contacts (*P* < 0⋅001). Furthermore, 24⋅6, 19⋅7 and 8⋅6 % of women who reported ≥8, 4–7 and 0–3 ANC contacts, respectively, reported supplement use while breastfeeding (*P* < 0⋅001). Associations between reported supplement use during pregnancy and lactation and maternal and household characteristics are shown in Table 4. In multivariable analyses, women with complete primary education were 2⋅4 times more likely to report supplement use during pregnancy compared to women with incomplete primary education (OR 2⋅35, 95 % CI 1⋅28, 4⋅30; *P* < 0⋅01). Compared to delivery at a hospital, women who delivered their child at home were less likely to report supplement use during pregnancy (OR 0⋅19, 95 % CI 0⋅09, 0⋅40; *P* < 0⋅001) and lactation (OR 0⋅35, 95 % CI 0⋅15, 0⋅83; *P* < 0⋅05). Women with grater gravidity, those of ethnic minority populations and those living in food insecure households were less likely to report use of supplements in the bivariate model, although this did not remain significant in the multivariable model.

**Table 4.** Factors associated with reported supplement use during pregnancy and lactation among biological mothers (*n* 681) in the bivariate and multivariable models

Variables	Bivariate model				Multivariable model^a^			
	Supplement use during pregnancy		Supplement use during lactation		Supplement use during pregnancy		Supplement use during lactation	
	OR	95 % CI	OR	95 % CI	OR	95 % CI	OR	95 % CI
Maternal age	0⋅96	0⋅93, 0⋅99*	0⋅99	0⋅96, 1⋅03	1⋅02	0⋅96, 1⋅08		
Maternal ethnic group								
Lao	Ref.		Ref.		Ref.			
Khmu	0⋅22	0⋅05, 0⋅95*	0⋅75	0⋅43, 1⋅31	0⋅62	0⋅12, 3⋅17		
Hmong	0⋅07	0⋅02, 0⋅28***	0⋅54	0⋅30, 0⋅97*	0⋅28	0⋅06, 1⋅39		
Maternal education								
Incomplete primary	Ref.		Ref.		Ref.		Ref.	
Complete primary	4⋅42	2⋅82, 6⋅92***	1⋅96	1⋅21, 3⋅17**	2⋅35	1⋅28, 4⋅30**	1⋅43	0⋅82, 2⋅49
Gravidity	0⋅76	0⋅68, 0⋅84***	0⋅98	0⋅87, 1⋅10	0⋅85	0⋅71, 1⋅03		
Location of delivery								
Hospital	Ref.		Ref.		Ref.		Ref.	
Health centre	1⋅56	0⋅73, 3⋅33	1⋅17	0⋅74, 1⋅84	1⋅71	0⋅70, 4⋅13	1⋅55	0⋅86, 2⋅79
Home	0⋅12	0⋅07, 0⋅23***	0⋅25	0⋅12, 0⋅52***	0⋅19	0⋅09, 0⋅40***	0⋅35	0⋅15, 0⋅83*
Other	0⋅76	0⋅20, 2⋅84	0⋅23	0⋅05, 1⋅02	0⋅93	0⋅21, 4⋅00	0⋅28	0⋅06, 1⋅39
Household SES index	2⋅26	1⋅72, 2⋅97***	1⋅36	1⋅12, 1⋅67**	1⋅43	0⋅94, 2⋅16	1⋅11	0⋅84, 1⋅47
HFIAS	0⋅95	0⋅91, 0⋅99*	0⋅92	0⋅87, 0⋅98**	1⋅02	0⋅95, 1⋅09	0⋅86	0⋅90, 1⋅02

CI, confidence interval; HFIAS, household food insecurity access scale; OR, odds ratio; SES, socioeconomic status.

a Variables with *P* < 0⋅1 at bivariate level were included in the multivariable logistic regression model.

* *P* < 0⋅05; **P < 0⋅01; ***P < 0⋅001.

### Maternal and child thiamine supplement use after hospital discharge

Of the 434 children discharged from the hospital, 173 (39⋅9 %) children and 316 (72⋅8 %) mothers were prescribed oral thiamine supplements by the hospital at the child's discharge, which was confirmed by the electronic medical records. Among those, adherence information at the 4-week follow-up interview after hospital discharge was obtained for 115 children and 215 mothers.

The majority of caregivers who confirmed they received thiamine supplements (*n* 103) reported giving the thiamine supplements to the child 5–6 days a week (*n* 101; 98⋅1 %). Only one (1⋅0 %) caregiver reported giving the supplements less than once per week as the mother reported that the child was better. Similarly, the majority of biological mothers who confirmed receiving thiamine supplements (*n* 193) reported taking the supplements 5–6 times a week (*n* 189; 97⋅9 %). Only two mothers reported taking the supplements ≤1 day a week. The reported reasons for non-compliance were that the supplements were not allowed with the postpartum dietary restrictions (*n* 1) and the woman did not like the smell of the supplement (*n* 1).

### Maternal supplement and condiment use and postpartum dietary restrictions

Table 5 shows the proportion of condiments habitually consumed by women. While salt consumption was reported on a daily basis by almost all women (99⋅4 %), fish sauce and soy sauce were regularly consumed by around half the women. Furthermore, of the women who regularly consumed the condiments ≥3–4 days a week, only 41⋅5 and 61⋅3 % continued to consume fish and soy sauce, respectively, when adhering to postpartum food avoidances, whereas the majority of women (98⋅7 %) continued consuming salt even while restricting other foods postpartum.

**Table 5.** Proportion of women (*n* 482) reporting the consumption of condiments habitually and when adhering to traditional postpartum dietary restrictions^a^

	Salt	Fish sauce	Soy sauce
	*n* (%)		
Habitual consumption			
Daily	479 (99⋅4)	164 (34⋅0)	197 (40⋅9)
5–6 days a week	1 (0⋅2)	15 (3⋅1)	15 (3⋅1)
3–4 days a week	0 (0)	20 (4⋅1)	30 (6⋅2)
1–2 days a week	0 (0)	28 (5⋅8)	29 (6⋅0)
Never	2 (0⋅4)	255 (52⋅9)	211 (43⋅8)
Consumed when adhering to postpartum dietary restrictions^b^			
Yes	456 (98⋅7)	78 (41⋅5)	141 (61⋅3)
No	6 (1⋅3)	110 (58⋅5)	89 (38⋅7)

a Includes biological mothers only.

b Among women habitually consuming condiment 3–4 days a week or more; *n* 18 habitual salt consumers, *n* 11 habitual fish sauce consumers and *n* 12 habitual soy sauce consumers did not follow any postpartum dietary restrictions and are not included.

Of the 417 women asked about supplement use, 360 (86⋅3 %) women reported that they would take supplements during the period of postpartum dietary restrictions if provided to them by health centres.

## Discussion

Among women residing in northern Lao PDR enrolled in this study, ANC and antenatal supplement use was suboptimal. In contrast, reported adherence to post-discharge hospital prescribed thiamine supplements was high among children treated for TDDs and their mothers. Salt fortification with micronutrients could be a viable strategy for increasing micronutrient intakes among women, as salt is habitually consumed by all women and continues to be consumed even during the period of traditional postpartum dietary restrictions.

Although ANC was utilised by women in this study during their pregnancy with the enrolled child, ANC was inadequately utilised according to the most recent WHO guidance. Only a quarter of women reported receiving ≥8 ANC contacts during their pregnancy as recommended by the WHO^(2)^, with the majority receiving 4–7 contacts and 9⋅8 % not receiving any ANC. Reported ANC coverage in the present study was greater than that in the most recent national Lao Social Indicator Survey (LSIS II) in 2017 for Luang Prabang province, in which 17⋅1 and 21⋅5 % of women reported ≥8 and no ANC contacts, respectively^(4)^. While this may be due to methodological differences, it may also indicate improvements in ANC coverage between LSIS II conducted in 2017 (where women were interviewed about a live birth in the previous 2 years) and the study period (2019–2020).

In agreement with a systematic review of factors affecting ANC utilisation in developing countries^(41)^ and the LSIS II^(4)^, the present study found that maternal education and higher household SES were associated with ANC attendance, while women with greater gravidity, home delivery and of ethnic minority populations were less likely to attend adequate ANC. In the present study, just 10⋅8 % of women belonging to the Hmong ethnic group reported ≥8 ANC contacts during their pregnancy, compared to 47⋅1 % of Lao women. Similarly, other studies have observed low ANC utilisation rates among women of ethnic minority populations in Lao PDR, especially among those with lower educational attainment^(42,43)^. Furthermore, ANC enhances hospital deliveries attended by skilled birth health workers, thereby reducing pregnancy and delivery-related morbidity and mortality. Hospital deliveries were the highest among women belonging to the Lao ethnic group in this study (48⋅1 %), while over a third of Hmong women gave birth at home. Language barriers, rural residence and greater distance to health facilities with a need for transportation can result in women of ethnic minorities not fully benefitting from ANC services^(43,44)^. Three quarters of the Laotian population live in remote rural areas, with a higher prevalence rate among minority ethnic groups, who thus have less access to services and infrastructure and higher rates of poverty and malnutrition^(45)^.

ANC is an opportunity to counsel women on the importance of nutrition and antenatal supplements. Indeed, women in the present study who reported ANC attendance were more likely to report use of antenatal supplements. However, similarly to ANC, antenatal supplement use was suboptimal during pregnancy and especially lactation, with 84⋅6 % of women reporting supplement use during their pregnancy and only 17⋅3 % while breastfeeding. This is despite 87⋅5 and 85⋅0 % of health facilities in Luang Prabang province that were interviewed as part of this study, reporting that supplements were provided during pregnancy and lactation, respectively. In Lao PDR, healthcare facilities are advised to distribute thiamine supplements to women during pregnancy and for 3 months postpartum in high-risk areas^(27,28)^ due to the recognised risk of TDD and associated mortality and morbidity among predominantly breastfed infants in the first year of life^(46)^. Despite 86⋅3 % of interviewed healthcare facilities in Luang Prabang province reporting the distribution of thiamine supplements to women postpartum, less than 10 % of women reported use of thiamine supplements while breastfeeding. A 2013–2014 World Bank survey of 120 health centres across Lao PDR also found a wide discrepancy between facilities offering micronutrient supplements during ANC and availability^(47)^, highlighting that supply constraints may be a factor in healthcare providers complying with the ANC guidelines, although this was not explored in the present study. It has previously been reported that only half of women in Lao PDR took any iron supplements during pregnancy and adherence was lowest among women belonging to the Hmong ethnic group (24 %), in rural areas without roads (22 %), among poor women (24 % in the poorest quintile) and women with no education (22 %)^(6)^. In agreement with this and other previous studies^(48,49)^, the present study found that higher education and household SES were associated with supplement use, while greater gravidity, home delivery, household food insecurity and women of ethnic minority populations were less likely to use supplements. Poor adherence is a barrier to the effectiveness of micronutrient supplementation programmes, even in settings with high coverage^(50)^, and may be due to low awareness and knowledge and a lack of understanding of the benefits due to insufficient counselling of pregnant women^(51–53)^. Health providers should, therefore, be sufficiently trained to confidently deliver health promotion messages^(45,54)^ and counsel women on the benefits of antenatal supplements to ensure better compliance throughout pregnancy and postpartum^(55,56)^. Further research should aim to understand why reported antenatal supplement use among these women is so low compared to the number of health centres reporting the distribution of supplements, and whether this is due to supply issues at the health centres, poor counselling or a lack of knowledge or awareness by women.

In contrast to antenatal supplementation, adherence to prescribed thiamine following discharge from the hospital for TDD-like symptoms was high among both children and mothers, with over 95 % reporting taking supplements five to six times a week. Adherence may be higher as this was a curative treatment, rather than a preventive measure. During pregnancy, little perceived health benefits of ANC or preventive supplementation, particularly if problems do not arise and women have not previously experienced problems, may deter women from taking antenatal supplements^(44,57,58)^. Thus, antenatal supplements may not be seen as necessary by women, whereas therapeutic thiamine supplements following hospitalisation are seen as essential to aid their child's recovery from illness.

ANC and supplementation utilisation may be low due to physical, geographical and financial factors but may also be influenced by cultural factors and traditional ideas and beliefs surrounding pregnancy and childbirth^(41,59)^. In Lao PDR, dietary restrictions and food avoidances are commonplace during the perinatal period, especially postpartum^(31,32)^, and it remains relatively unexplored if supplements are culturally acceptable during this restrictive period. A high proportion of women (86⋅3 %) in the present study reported that they would be willing to take supplements during the period of postpartum dietary restrictions if provided to them by health facilities, indicating that these may be culturally acceptable. However, as previously discussed, ≤18 % of women reported taking some type of supplement while breastfeeding despite a high proportion of health facilities reporting the distribution of supplements. While women may have over-reported potential supplement use, the social desirability score, an indication of a respondent answering questions viewed as favourably by society, was low. Thus, it would be beneficial to further understand this discordance between reported adherence and stated willingness, and if it is due to a lack of knowledge and awareness of the benefits of micronutrient supplements.

Food fortification provides an additional, sustainable and population-wide public health intervention that can ensure equitable delivery of micronutrients with limited behaviour change, providing that an appropriate vehicle is selected^(60)^. Fish and soy sauces are widely consumed across Asia due to their availability and affordability and are established vehicles for iron and iodine fortification in Cambodia^(61,62)^, Vietnam^(63,64)^ and Thailand^(65)^. Additionally, experimental thiamine fortified fish sauces significantly increased thiamine status among Cambodian women and their children^(66,67)^. However, this study indicated that fish and soy sauces may not be suitable fortification vehicles in northern Lao PDR as they were only consumed regularly (defined as 3–4 days a week or more) by approximately half of the women participating in the study. Moreover, women who regularly consumed these condiments reported restricting these postpartum when adhering to food avoidances. Conversely, salt was habitually consumed by the vast majority of women and rarely restricted postpartum, suggesting that this may be a viable fortification vehicle that would reach a significant proportion of women and improve micronutrient status both pre- and postnatally. Currently, iodine and thiamine co-fortification of salt has not been tested, and a multiple micronutrient fortified salt, including thiamine, evaluated as part of an Indian school feeding programme did not assess thiamine status^(68)^. A study among lactating women in Cambodia found that, based on estimated salt intakes, a fortification dose of 275 mg thiamine/kg salt could increase thiamine intakes to 1⋅2–4⋅3 mg/day, meeting recommended intakes for thiamine from fortified salt alone^(69)^. There are no known adverse effects of high thiamine intakes and thus no tolerable upper intake level has been set^(70)^; hence, thiamine fortification programmes should be able to be safely implemented alongside antenatal supplementation accompanied by measures of monitoring thiamine intakes and status. Additionally, salt fortification programmes are compatible with global salt reduction strategies^(71)^, providing regular evaluation of usual salt intakes to ensure fortification levels adequately and safely meet population needs. Further research should consider multiple micronutrient fortified salt, including stability, sensory characteristics, cost and price implications, as a means of delivering micronutrients beyond iodine^(72)^.

Strengths of this study include the large sample size and the inclusion of a range of variables at the individual and household level. As women were interviewed about pregnancies within the 18 months prior to study enrolment, recall bias is a limitation of this study. Additionally, this study was a secondary analysis of a hospital-based prospective cohort study including a frequency-matched community-based comparison group in Luang Prabang and surrounding provinces, and thus, the study population was not representative of northern Lao PDR, nor is it generalisable to other geographical regions or ethnic groups of Lao PDR. Despite these limitations, this analysis does contribute to the understanding of ANC and antenatal supplement utilisation among women in northern Lao PDR, although qualitative analysis would allow for further in-depth exploration of barriers to ANC and supplement use. Additionally, it is important to understand the coverage of antenatal supplements (IFA and thiamine) across different provinces and regions of Lao PDR and the underlying regions for any disparities, to ensure antenatal supplementation programmes are adequately implemented.

## Conclusion

Despite positive trends in maternal health service indicators^(73)^, suboptimal utilisation of ANC and antenatal supplements remains among women in northern Lao PDR, particularly among women of ethnic minority populations, low household SES and those with lower educational attainment. Understanding individual, socioeconomic, cultural and behavioural predictors and barriers of ANC and antenatal supplement use can aid the design and targeting of public health strategies to increase adherence. Alongside targeted antenatal supplementation, salt fortification with micronutrients may be a viable population-based intervention that needs further evaluation.

## Supplementary material

For supplementary material accompanying this paper visit https://doi.org/10.1017/jns.2022.94 click here to view supplementary material
