# Supplementary material for: Nutrition and health-seeking practices during pregnancy and lactation and potential strategies to increase micronutrient intakes among women in northern Lao PDR
Source: J Nutr Sci. 2022 Oct 28;11:e95. doi: 10.1017/jns.2022.94 (PMC9641509; doi:10.1017/jns.2022.94)
Supplement: Supplementary file 1 [file jnssup.zip › S2048679022000945sup001.docx]

**Supplementary Table S1.** Inclusion and exclusion criteria for infants and young children and their mothers

| **Study group** | **Inclusion criteria** | **Exclusion criteria** |
| --- | --- | --- |
| Hospitalised children | Target age range: 21 days to <18 mon  Informed parental consent  Plus at least one of the following signs and symptoms suggestive of TDD :  Enlarged liver (>2 cm below right costal margin, supine exam)  Edema  Tachypnea (RR >60/min for 3-8 wks; >50/min for 2-11 mon; >40/min for 12-18 mon)  Tachycardia (HR >160/min for <12 mon; >120/min for 12-18 mon)  Oxygen saturation <92%  Difficulty breathing  Refusal of breastmilk/food for >24 hours  Repetitive/recurrent vomiting (≥3 times in 24 hours)  Persistent crying with no obvious cause  Hoarse voice/loss of voice  Nystagmus/abnormal eye movements  Muscle twitching  Loss of consciousness  Convulsion  Opisthotonos/abnormal posturing  Acute/flaccid paralysis | No specific exclusion criteria |
| Community children | Frequency matched to hospitalised children based on age, sex and village of residence  Informed parental consent | Severe acute illness warranting hospital referral |
| Mothers^*^ | Informed consent | Severe acute illness warranting hospital referral  Unable to provide informed consent due to reduced decision-making ability |

HR, heart rate; RR, respiratory rate; TDD, thiamine deficiency disorders

^*^ All mothers or other female primary caregivers of enrolled children in the hospital and community were eligible for participation in the study
