# Supplementary material for: Nutrition and health-seeking practices during pregnancy and lactation and potential strategies to increase micronutrient intakes among women in northern Lao PDR
Source: J Nutr Sci. 2022 Oct 28;11:e95. doi: 10.1017/jns.2022.94 (PMC9641509; doi:10.1017/jns.2022.94)
Supplement: Supplementary file 1 [file jnssup.zip › S2048679022000945sup002.docx]

**Supplementary Table S2.** Characteristics of women by ethnic group^*^

| **Characteristics** | **Lao**  **(n = 104)** | **Khmu**  **(n = 228)** | **Hmong**  **(n = 334)** | **Other**  **(n = 25)** | ***P* values** |
| --- | --- | --- | --- | --- | --- |
|  | **n (%) or mean ± SD** | | | |  |
| Age (yrs) | 26.0 ± 5.2 | 24.8 ± 6.5 | 24.4 ± 6.8 | 24.7 ± 6.8 | 0.16 |
| Education |  |  |  |  | <0.001 |
| No formal education | 3 (2.9) | 29 (12.7) | 100 (30.0) | 3 (12.0) |  |
| Some/completed primary | 19 (18.3) | 92 (40.4) | 94 (28.2) | 10 (40.0) |  |
| Some completed secondary | 58 (55.8) | 97 (42.5) | 130 (39.0) | 10 (40.0) |  |
| College/university | 24 (23.1) | 10 (4.4) | 9 (2.7) | 2 (8.0) |  |
| Gravidity^†^ | 1.9 ± 0.8 | 2.3 ± 1.4 | 3.0 ± 2.1 | 2.0 ± 1.2 | <0.001 |
| ANC contacts |  |  |  |  | <0.001 |
| 0 – 3 | 8 (7.7) | 46 (20.5) | 167 (51.5) | 3 (12.0) |  |
| 4 – 7 | 47 (45.2) | 104 (46.4) | 122 (37.7) | 13 (52.0) |  |
| ≥8 | 49 (47.1) | 74 (33.0) | 35 (10.8) | 9 (36.0) |  |
| Location of delivery of infant |  |  |  |  | <0.001 |
| Home | 5 (4.8) | 51 (22.5) | 119 (35.7) | 2 (8.0) |  |
| Health centre | 45 (43.3) | 125 (55.1) | 103 (30.9) | 11 (44.0) |  |
| Hospital | 50 (48.1) | 33 (14.5) | 101 (30.3) | 11 (44.0) |  |
| Other | 4 (3.8) | 18 (7.9) | 10 (3.0) | 1 (4.0) |  |
| Reported supplement use during pregnancy | 102 (98.1) | 207 (90.8) | 246 (73.9) | 23 (92.0) | <0.001 |
| Reported supplement use during lactation | 26 (25.0) | 46 (20.2) | 41 (12.3) | 5 (20.0) | 0.009 |
| Household SES index^‡^ | 1.2 ± 0.8 | -0.2 ± 0.9 | -0.3 ± 0.8 | 0.7 ± 0.8 | <0.001 |
| Food insecurity category^§^ |  |  |  |  | <0.001 |
| Moderate to severe | 8 (7.7) | 98 (43.0) | 123 (36.9) | 5 (20.0) |  |
| Mild to none | 96 (92.3) | 130 (57.0) | 210 (63.1) | 20 (80.0) |  |
| Social desirability score^\|^ | 2.8 ± 1.0 | 2.7 ± 1.0 | 2.8 ± 1.2 | 2.8 ± 1.4 | 0.78 |
| Social desirability category |  |  |  |  | 0.004 |
| Low | 32 (30.8) | 77 (33.8) | 111 (33.2) | 9 (36.0) |  |
| Medium | 53 (51.0) | 114 (50.0) | 128 (38.3) | 9 (36.0) |  |
| High | 16 (15.4) | 34 (14.9) | 84 (25.1) | 4 (16.0) |  |
| Very high | 3 (2.9) | 3 (1.3) | 11 (3.3) | 3 (12.0) |  |
| BMI (kg/m^2^) |  |  |  |  | 0.97 |
| <18.5 | 10 (9.7) | 16 (7.3) | 29 (8.9) | 2 (8.0) |  |
| ≥18.5 – 24.9 | 79 (76.7) | 173 (78.6) | 252 (77.3) | 19 (76.0) |  |
| ≥25.0 – 29.9 | 12 (11.7) | 28 (12.7) | 43 (13.2) | 4 (16.0) |  |
| ≥30.0 | 2 (1.9) | 3 (1.4) | 2 (0.6) | 0 (0.0) |  |
| Height <150 cm^¶^ | 25 (24.3) | 108 (49.1) | 223 (68.4) | 7 (28.0) | <0.001 |
| MUAC <23.5 cm^**^ | 36 (34.6) | 89 (40.1) | 144 (44.0) | 17 (68.0) | 0.017 |

ANC, antenatal care; BMI, body mass index; MUAC, mid-upper arm circumference; SES, socioeconomic status

^*^ Sample size for different assessments may vary; ANC contacts during pregnancy with the study child and gravidity are for biological mothers; anthropometry and haemoglobin were only assessed for biological and adoptive mothers

^†^ n = 104 Lao; n = 226 Khmu; n = 332 Hmong; n = 25 other

^‡^ SES index derived from principal component analysis using self-reported measures of housing characteristics, household access to utilities and household ownership of assets and land; n = 103 Lao; n = 226 Khmu; n = 333 Hmong; n = 25 other

^§^ Food insecurity assessed using the Household Food Insecurity Access Scale

^|^ A social desirability score was created by adding up the number of socially desirable responses: 0-2 = low score; 3 = medium score; 4 = high score; 5 = very high score

^¶^ n = 103 Lao; n = 220 Khmu; n = 326 Hmong; n = 25 other

^**^ n = 104 Lao; n = 222 Khmu; n = 327 Hmong; n = 25 other
