# Supplementary material for: Nutrition and health-seeking practices during pregnancy and lactation and potential strategies to increase micronutrient intakes among women in northern Lao PDR
Source: J Nutr Sci. 2022 Oct 28;11:e95. doi: 10.1017/jns.2022.94 (PMC9641509; doi:10.1017/jns.2022.94)
Supplement: Supplementary file 1 [file jnssup.zip › S2048679022000945sup003.docx]

**Supplementary Table S3.** Reported supplement distribution to pregnant and lactating women by health centres, district and provincial hospitals across 6 provinces in northern Lao PDR

|  | **Luang Prabang province**  **n (%)** | **Other provinces^*^**  **n (%)** |
| --- | --- | --- |
| Health care facilities interviewed | 80 (82.5) | 17 (17.5) |
| Supplements distributed to pregnant women^†^ | | |
| IFA only | 8 (10.0) | 11 (64.7) |
| IFA and thiamine | 70 (87.5) | 4 (23.5) |
| Iron alone only | 0 (0.0) | 0 (0.0) |
| Iron alone, folic acid alone and thiamine | 2 (2.5) | 0 (0.0) |
| Folic acid alone only | 0 (0.0) | 1 (5.9) |
| Folic acid alone and thiamine | 0 (0.0) | 1 (5.9) |
| Supplements distributed to lactating women^†^ | | |
| IFA only | 10 (12.5) | 9 (52.9) |
| IFA and thiamine | 68 (85.0) | 6 (35.3) |
| Iron alone only | 1 (1.3) | 0 (0.0) |
| Iron alone, folic acid alone and thiamine | 1 (1.3) | 0 (0.0) |
| Folic acid alone only | 0 (0.0) | 1 (5.9) |
| Folic acid alone and thiamine | 0 (0.0) | 1 (5.9) |
| Thiamine supplements distributed during pregnancy^‡^ | 72 (90.0) | 5 (29.4) |
| Thiamine supplements distributed during lactation^§^ | 69 (86.3) | 7 (41.2) |

IFA, iron-folic acid

^*^ Other provinces: Oudomxay (n = 7); Xayaboury (n = 6); Phongsaly (n = 2); Luangnamtha (n = 1); Bokeo (n = 1)

^†^ Dosages reported by health care facilities: Iron-folic acid: 60 mg iron + 400 µg folic acid (n = 55 health facilities); 200 mg iron + 400 µg folic acid (n = 38 health facilities); Iron alone: 200 mg (n = 2 health facilities); Folic acid alone: 5 mg (n = 2 health facilities); 4 mg (n = 1 health facility); 400 µg (n = 1 health facility); Thiamine: 100 mg (n = 77 health facilities)

^‡^ Supplements reportedly distributed during the first antenatal care contact

^§^ n = 4 health care facilities distributed thiamine supplements for 1 month postpartum; n = 72 health care facilities distributed thiamine supplements for 3 months postpartum
